# A metatranscriptomics strategy for efficient characterization of the microbiome in human tissues with low microbial biomass

**DOI:** 10.1080/19490976.2024.2323235

**Published:** 2024-02-29

**Authors:** Joana Pereira-Marques, Rui M. Ferreira, Ceu Figueiredo

**Affiliations:** a i3S – Instituto de Investigação e Inovação em Saúde, Universidade do Porto, Porto, Portugal; b Ipatimup – Institute of Molecular Pathology and Immunology of the University of Porto, Porto, Portugal; c Department of Pathology, Faculty of Medicine of the University of Porto, Porto, Portugal

**Keywords:** Microbiome, metatranscriptomics, low microbial biomass, mucosal tissues, bacteria, mock bacterial community, computational biology

## Abstract

The high background of host RNA poses a major challenge to metatranscriptome analysis of human samples. Hence, metatranscriptomics has been mainly applied to microbe-rich samples, while its application in human tissues with low ratio of microbial to host cells has yet to be explored. Since there is no computational workflow specifically designed for the taxonomic and functional analysis of this type of samples, we propose an effective metatranscriptomics strategy to accurately characterize the microbiome in human tissues with a low ratio of microbial to host content. We experimentally generated synthetic samples with well-characterized bacterial and host cell compositions, and mimicking human samples with high and low microbial loads. These synthetic samples were used for optimizing and establishing the workflow in a controlled setting. Our results show that the integration of the taxonomic analysis of optimized Kraken 2/Bracken with the functional analysis of HUMAnN 3 in samples with low microbial content, enables the accurate identification of a large number of microbial species with a low false-positive rate, while improving the detection of microbial functions. The effectiveness of our metatranscriptomics workflow was demonstrated in synthetic samples, simulated datasets, and most importantly, human gastric tissue specimens, thus providing a proof of concept for its applicability on mucosal tissues of the gastrointestinal tract. The use of an accurate and reliable metatranscriptomics approach for human tissues with low microbial content will expand our understanding of the functional activity of the mucosal microbiome, uncovering critical interactions between the microbiome and the host in health and disease.

## Introduction

The microbiome refers to the collection of microorganisms that inhabit a well-defined environment, including bacteria, archaea, fungi, microbial eukaryotes, and viruses, but also to their genetic information, structural elements, and metabolites, and to the environmental conditions of the habitat they occupy.^1^ The gut microbiome has a well-recognized role in the maintenance of human physiology and health, but also has a significant impact on the development of multiple diseases, including diseases of the gastrointestinal (GI) tract such as inflammatory bowel disease and cancer.^2–4^ Our understanding of the composition and functional potential of the gut microbiome has largely stemmed from studies of the fecal microbial communities.^5,6^ However, the fecal microbiome does not fully reflect the microbial communities at the mucosal interfaces of the GI tract,^7^ thus limiting a comprehensive view of the functional role of the local microbiota.

Microbial characterization methods such as 16S ribosomal RNA (rRNA) gene and whole metagenome sequencing only describe the microorganisms and genes present in the community and do not provide information on microbial cell viability and transcriptional activity.^8^ In contrast, metatranscriptomics enables to explore the functional and metabolic capabilities of the microbiome.^9^ Metatranscriptomics uses shotgun sequencing of the RNA to profile the collective microbial transcriptome, identifying microbial expressed genes and associated functions, and the metabolically active members of the community.^9,10^ Nevertheless, it remains underutilized due to experimental and analytical limitations, including the short half-life of messenger RNA (mRNA),^10,11^ the high abundance of rRNA,^12,13^ the absence of poly(A)-tail in the mRNA of most prokaryotes, which makes selective capture of microbial transcripts challenging,^10,14^ and the reliance on bioinformatic tools developed for metagenomics and single-organism transcriptomics, rather than for metatranscriptomics.^10,15^

Human samples pose a major challenge in metatranscriptomics, in particular when there is high background of host RNA.^10^ The amount of host content and microbial biomass varies considerably across sample type and body sites. For example, stool samples have high microbial load, while human tissue specimens contain lower microbial load and a low ratio of microbial to host cells.^9,16,17^ The latter require stringent precautions during sample collection, processing, and data analysis (e.g.: inclusion of multiple controls), as microbial contamination can easily outcompete the biological signal within the samples.^17,18^ In samples with a low ratio of microbial to host cells, sequencing capacity is largely used on host RNA. Thus, high depths are required to maximize detection of microbial sequences, making such studies costly and computationally intensive.^15^ Importantly, there is no computational workflow specifically tailored for the taxonomic and functional analysis of the metatranscriptome of this type of samples. Due to the aforementioned limitations, metatranscriptomics has mainly been applied to microbe-rich samples,^5,19,20^ while its application in tissues with lower microbial biomass has yet to be explored. To address this knowledge gap, our aim was to develop an effective metatranscriptomics strategy that can accurately characterize the microbiome in human tissues with low ratio of microbial to host cells, such as mucosal specimens of the GI tract.

## Results

### Generation of synthetic host-microbiome samples, metatranscriptomics workflow, and pre-processing of the metatranscriptomics data

**Figure 1.**
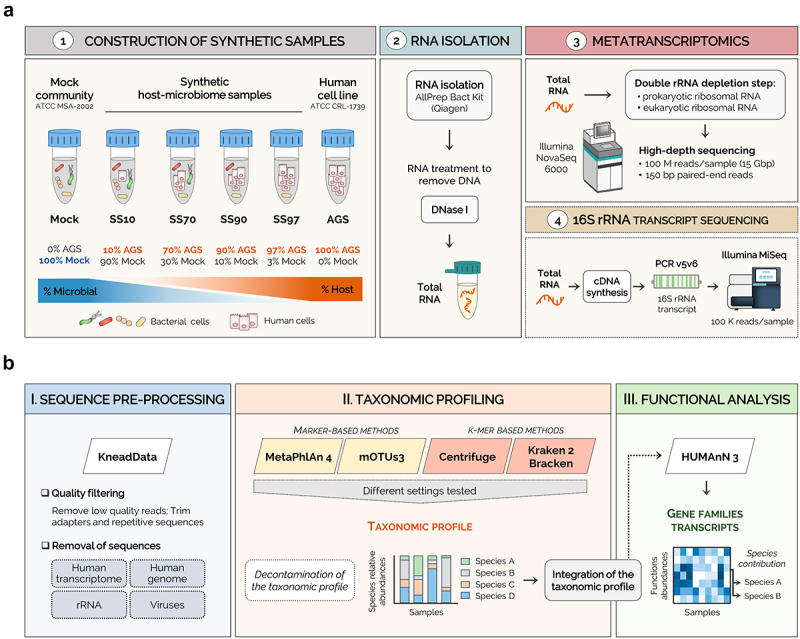
Metatranscriptomics workflow for synthetic host-microbiome samples.

We experimentally obtained four sets of standards mimicking human samples with distinct host to microbial cell ratios. For that, a mock community (Mock) of bacterial cells (Supplementary Table S1) was spiked with eukaryotic cells of a human cell line, to generate synthetic samples with 10%, 70%, 90%, and 97% host cells (SS10, SS70, SS90, and SS97, respectively). Total RNA from freshly prepared synthetic samples was immediately isolated and treated with DNase, to guarantee high yield and quality. For metatranscriptomics, both prokaryotic and eukaryotic rRNA were depleted to enrich for the mRNA fraction. Sequencing was performed in the Illumina NovaSeq 6000 with a high depth to improve the sensitivity of microbiome analysis. In parallel, 16S rRNA transcript profiling was conducted from total RNA samples (Figure 1a).

(a) To establish and optimize our metatranscriptomics strategy, four synthetic samples mimicking human samples with distinct host to bacterial cell ratios were created to contain 10%, 70%, 90%, and 97% host cells (SS10, SS70, SS90, and SS97, respectively) by spiking the mock microbial community (100% bacterial cells; Mock) with a human cell line (100% host cells; AGS). To guarantee high RNA yield and quality, the freshly prepared synthetic samples were immediately processed. Total RNA was isolated and treated with DNase I to remove contaminant DNA. For metatranscriptomics, due to the high predominance of rRNAs in total RNA, a double depletion of prokaryotic and eukaryotic rRNA was performed. All samples were sequenced on the Illumina NovaSeq 6000 platform at high depth (15 Gbp). In parallel, next-generation sequencing of the 16S rRNA transcript was performed from total RNA samples. (b) The raw metatranscriptome sequencing data were pre-processed to trim the sequencing adapters, and to remove low-quality reads and rRNA, viruses, and human sequences. Then, mapping-based methods were used for taxonomic and functional analysis of the metatranscriptomes. First, the taxonomic profiling was performed using different computational tools submitted with multiple settings to improve species identification. After *in silico* removal of potential taxa contaminants, the decontaminated taxonomic profiles from each classifier were integrated into the functional analysis performed by HUMAnN 3, which stratifies community functional profiles according to contributing species.

For establishing the computational workflow, the raw metatranscriptome sequencing data were pre-processed to trim the sequencing adapters, and to remove low-quality reads and rRNA, viruses, and host sequences. Taxonomic profiling was performed using different classifiers submitted with multiple settings to improve species identification (Table 1). After *in silico* removal of taxa contaminants, the decontaminated taxonomic profiles were integrated into HUMAnN 3 functional analysis, which stratifies community functional profiles according to the contributing species (Figure 1b).

**Table 1.** The settings used with different parameters for each taxonomic classifier to improve the identification of species.

Taxonomic classifier	Settings	Parameters
*MetaPhlAn 4*	Default	-stat_q 0.2; -min_mapq_val 5
	Settings 1	-stat_q 0.1; -min_mapq_val 5
	Settings 2	-stat_q 0.05; -min_mapq_val 5
	Settings 3	-stat_q 0.05; -min_mapq_val −1
*mOTUs3*	Default	-g3
	Settings 4	-g2
	Settings 5	-g1
*Centrifuge*	Default	-min-hitlen 22; -k 5
*Kraken 2/Bracken*	Default	-confidence 0
	Settings 6	-confidence 0.05

The five metatranscriptome datasets yielded a large number of raw single-end reads, ranging from 102 to 132 million, which is consistent with the expected high sequencing depth per sample (~15 Gbp) (Supplementary Table S2). The proportion of quality-filtered reads was high across all samples (94% to 96%). As anticipated, after sequence pre-processing, the number of reads consistently decreased in synthetic samples with increasing proportions of host content (Supplementary Table S2). The overall number of rRNA and viral sequences discarded was relatively low (Supplementary Table S3), showing that the final number of reads observed in SS70, SS90, and SS97 were due to the removal of human sequences. Overall, these results validate the experimental generation of host-microbiome synthetic samples.

### Optimized Kraken 2/Bracken generates the most accurate taxonomic profile of synthetic samples with low ratio of microbial to host cells

To determine the most effective taxonomic classifier to profile the metatranscriptome of low microbial biomass samples, we compared the microbial profiles of all samples using MetaPhlAn 4, mOTUs3, Centrifuge, and Kraken 2/Bracken, with different parameter settings (Table 1). Classifiers’ performance was evaluated by the number of bacteria of the mock identified by each tool and the number of false-positives detected (Others), regardless of their abundances. This takes into consideration the fact that the evenness in proportion of bacterial cells in the mock does not necessarily correspond to an even relative abundance of microbial transcripts.

**Figure 2.**
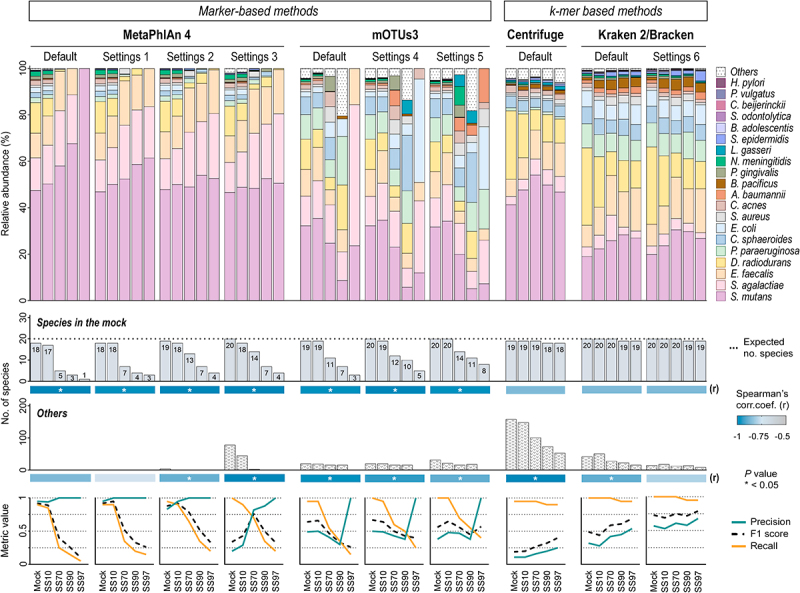
Kraken 2/Bracken with optimized settings accurately profiles the metatranscriptome of synthetic samples with low microbial biomass.

The identification of the 20 bacterial species in the Mock sample was achieved by all methods, except for Centrifuge; Kraken 2/Bracken detected all species using the default settings, while MetaPhlAn 4 and mOTUs3 only accomplished this with less stringent parameters (Figure 2).

Taxonomic analysis at the species-level of the metatranscriptome of synthetic samples (SS) using MetaPhlan 4, mOTUs3, Centrifuge, and Kraken 2/Bracken with different parameter settings. Upper color-coded bar plot showing the relative abundances of the 20 bacterial species of the mock community and of other microbial species that are not present in the mock community (Others) identified by each classifier. Middle bar plots showing the number of bacterial species of the mock community each tool was able to identify and the number of false-positive species detected (Others). Dotted line shows the expected number of species of the mock community in the samples. Spearman’s rank correlation between the number of species identified in the synthetic samples and their percentage of host cells was determined for each classifier. The performance metrics precision, recall, and F1 score for the classifications given by each classifier in the synthetic samples are shown in the bottom panel. The complete names of the species of the mock community are shown in the Supplementary Table S1. * stands for statistically significant at *P* < .05.

The ability of the marker-based methods MetaPhlAn 4 and mOTUs3 to identify the species of the mock community gradually decreased with the increase in host content of synthetic samples, with inverse correlations for all settings (Spearman’s *r* = −0.975 or *r* = −1, *p* < .005; Figure 2). Therefore, the recall for these methods was much higher in the Mock and SS10 than in the synthetic samples with 70%, 90%, and 97% host cells. The reduction in sensitivity was more striking in SS97, especially when using the default settings (recall 0.05 or 0.15; Figure 2). The use of other settings did not markedly improve microbial classification; less stringent settings in MetaPhlAn 4 slightly improved recall in all samples, but enhanced false-positives, especially in Mock and SS10. This was more pronounced in settings 3, leading to strong decreases in precision in these samples (Figure 2). The performance of mOTUs3 was broadly similar between settings, with high recall and low precision in Mock and SS10, and the opposite in SS97, due to the absence of false-positives. Overall, marker-based methods had good performance to profile the metatranscriptome of samples with high bacterial load, but were less effective in samples with lower microbial content.

The *k*-mer based methods Centrifuge and Kraken 2/Bracken were the most sensitive classifiers, with a recall between 0.9 and 1 across all samples. Although Centrifuge had high recall in all sample types, it showed a high trade-off between recall and precision, generating a large number of false-positive species, ranging from fifties (in SS97) to hundreds (in Mock). These results explain the lowest precision values of Centrifuge (<0.26) compared with other classifiers, and the F1 scores below 0.41 (Figure 2).

Kraken 2/Bracken was the classifier with the highest recall in samples with low microbial content, such as SS70, SS90, and SS97. To decrease the false-positive classifications of Kraken 2/Bracken, which were impairing its precision values (0.28 to 0.54), we optimized the confidence threshold of Kraken 2 (Settings 6). As a result, precision was maximized without changing recall, especially in SS97 (precision of 0.68). Thus, Kraken 2/Bracken with optimized settings generated the most accurate taxonomic profile (highest F1 score) of SS97, the synthetic sample with 97% host cells and very low microbial content.

As seen in Figure 2, none of the marker- or *k*-mer-based methods detected one of the species of the mock (*Helicobacter pylori*) in the metatranscriptomes of synthetic samples with very high host content (SS90 and SS97). In fact, when we analyzed the pre-processed reads of SS90 and SS97 against the *H. pylori* 26695 genome (GenBank AE000511.1, ATCC 700392), we could not identify any *H. pylori* reads, while the same analysis for SS70 detected 41 *H. pylori* reads (data not shown), thus suggesting that this species is not detected by the different classifiers due to the absence of *H. pylori* transcripts in SS90 and SS97 synthetic samples. This may be due to the low transcriptional activity of the bacterium or to biases introduced during the experimental procedure from whole-cell material to sequenced RNA.

**Figure 3.**
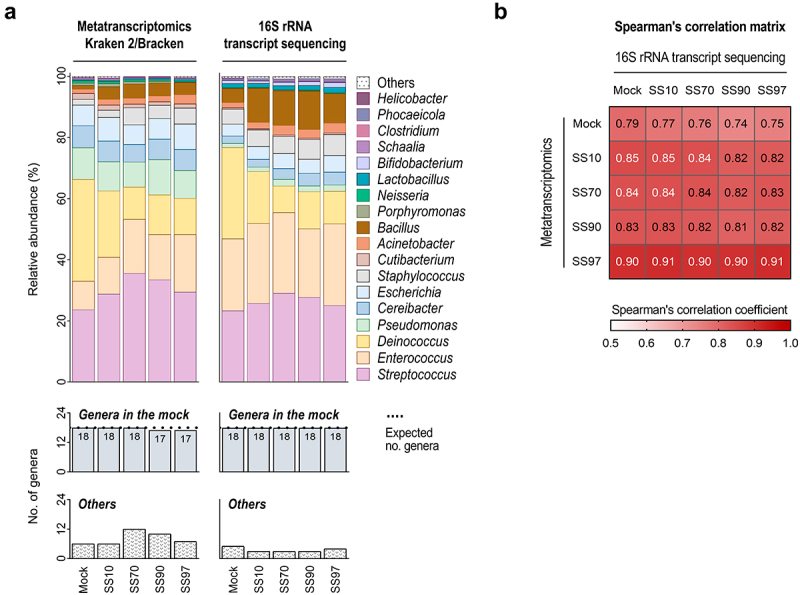
16S rRNA transcript sequencing validates the metatranscriptome profiles of Kraken 2/Bracken with optimized settings.

To compare the metatranscriptome profiles of Kraken 2/Bracken with optimized settings, we conducted 16S rRNA taxonomic analysis on the total RNA of synthetic samples (Figure 3, Supplementary Figure S1). For comparability between methods, the analysis was performed at the genus level, and the unassigned taxa in the 16S rRNA-based profiles (genera from the mock community classified at higher taxonomic ranks) were removed from the analysis (Supplementary Figure S1). 16S rRNA transcript sequencing successfully identified the 18 bacterial genera (100%) that compose the mock community (of 20 species) in all synthetic samples (Figure 3a). This method was also concordant regarding the relative abundance of transcriptionally active taxa seen in Kraken 2/Bracken metatranscriptome profiles (Figure 3a). Significant positive correlations between the taxonomic profiles of 16S rRNA transcript sequencing and metatranscriptomics with optimized Kraken 2/Bracken were observed (0.74 to 0.91, *p* < .001; Figure 3b and Supplementary Table S4). Overall, these data validate the metatranscriptome profiles of Kraken 2/Bracken with optimized settings.

(a) Taxonomic analysis at the genus-level of synthetic samples (SS) using metatranscriptomics with optimized Kraken 2/Bracken (left panel) and 16S rRNA transcript sequencing (right panel). Upper color-coded bar plots showing the relative abundances of the 18 bacterial genera of the mock community and of other microbial genera that are not present in the mock community (Others) identified by each method. Bar plots showing the number of bacterial genera of the mock community each method was able to identify and the number of false-positive genera detected (Others). Dotted line shows the expected number of genera of the mock community in the samples. (b) Spearman’s correlation matrix between the taxonomic profiles of metatranscriptomics with optimized Kraken 2/Bracken and 16S rRNA transcript sequencing from synthetic host-microbiome samples. Significance was considered for *p* < .05 (Supplementary Table S4).

Taken together, our results evidence Kraken 2/Bracken as the best-performing classifier to profile the metatranscriptome of samples with low microbial load and high percentage of host content.

### Optimized Kraken 2/Bracken has high sensitivity to profile the metatranscriptome in low microbial biomass simulated datasets

**Figure 4.**
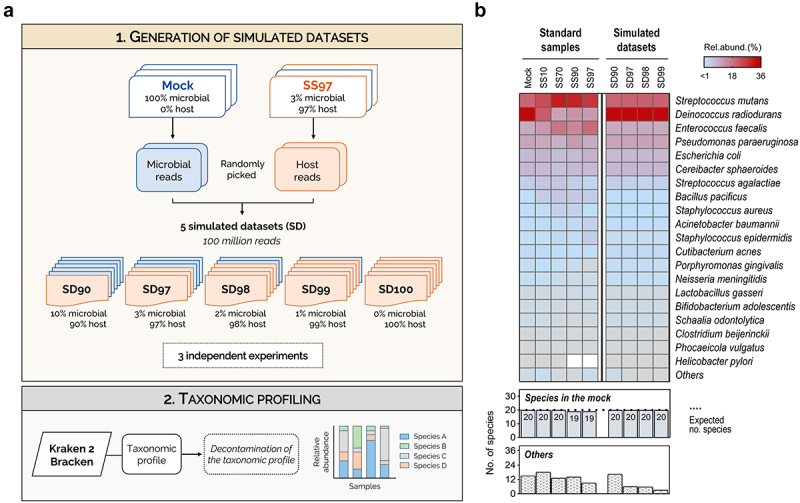
Kraken 2/Bracken with optimized settings has high sensitivity to profile the metatranscriptome in low microbial biomass simulated datasets.

To confirm that optimized Kraken 2/Bracken is the most effective classifier for metatranscriptomics of samples with low ratio of microbial to host cells, we next evaluated the ability of this method to identify all species from the mock community in simulated datasets with progressively higher proportion of host sequences. For that, microbial and host reads were randomly selected from our sequenced datasets (Mock and SS97, respectively), and were combined in different proportions at a fixed high depth, to create five simulated datasets (SD) containing 90%, 97%, 98%, 99%, and 100% host reads (SD90, SD97, SD98, SD99, and SD100, respectively). For each simulated dataset, three independent replicates were randomly generated and their taxonomic profiles were determined (Figure 4a and Supplementary Figure S2).

(a) Schematic representation of the experimental design to generate simulated datasets (SD) of the metatranscriptomes of synthetic samples with low microbial biomass. Microbial and host single-end reads were randomly selected from the mock community (Mock) and from the SS97 pre-processed datasets, and were combined in different proportions, at a fixed sequencing depth of 100 million reads, to generate five simulated datasets with progressively higher host sequences (90%, 97%, 98%, 99%, and 100%). Then, the taxonomic profiling of the simulated datasets was determined using Kraken 2/Bracken with optimized settings. (b) Taxonomic analysis at the species-level of the metatranscriptomes of synthetic samples (SS; left panel) and simulated datasets (SD; right panel) using Kraken 2/Bracken with optimized settings. Heat map showing the relative abundances of the 20 bacterial species of the mock community and of other microbial species that are not present in the mock community (Others). Data from simulated datasets is shown as the mean relative abundance of species from 3 independent experiments. Bar plots showing the number of bacterial species of the mock community that Kraken 2/Bracken with optimized settings was able to identify and the number of false-positive species detected (Others) on synthetic samples (left panel) and simulated datasets (right panel). Dotted line shows the expected number of species of the mock community in the samples.

Contrasting to the results in SS90 and SS97 where only 19 species were detected (likely due to the absence of transcripts of *H. pylori*), in the simulated datasets containing 90% and 97% host reads, all 20 species were successfully identified by Kraken 2/Bracken with optimized settings (Figure 4b). Furthermore, when the analysis was extended to simulated datasets with even lower microbial sequences (SD98 and SD99), optimized Kraken 2/Bracken also performed considerably well, detecting all species of the mock community and a low number of false-positives (Others; Figure 4b). Reinforcing the good performance of Kraken 2/Bracken, no statistically significant differences were observed between the taxonomic profiles of simulated datasets with increasing proportions of host reads (SD90 to SD99; Supplementary Table S5). As expected, in the SD100 which only contains human reads, no bacterial species were identified (Supplementary Figure S2).

Overall, these results support our previous analysis in synthetic samples, demonstrating the effectiveness of using optimized Kraken 2/Bracken to profile the metatranscriptome of samples with low ratio of microbial to host cells, even when the host content is above 97%.

### HUMAnN 3 functional analysis is improved with Kraken 2/Bracken microbial profile in synthetic samples with low microbial biomass

For functional analysis of the metatranscriptomes we used HUMAnN 3, which implements a tiered search; it involves an initial nucleotide search against the pangenomes of the species identified in the taxonomic pre-screening, followed by a translated search against the UniRef90 protein database for unclassified reads.^21^

**Figure 5.**
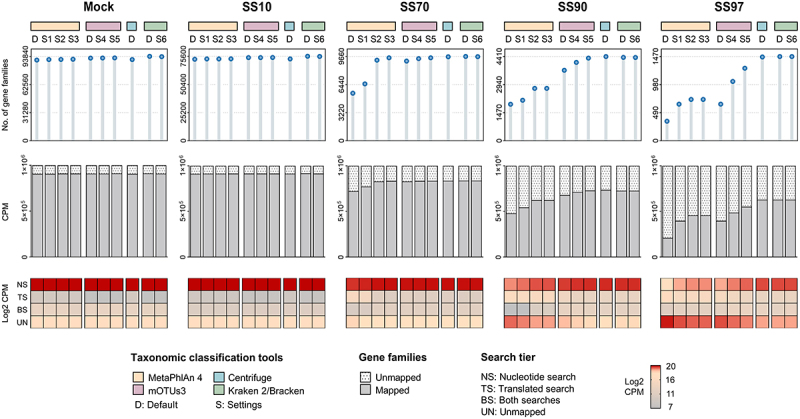
Kraken 2/Bracken improves HUMAnN 3 functional analysis of the metatranscriptome of synthetic samples with low microbial biomass.

To investigate the effectiveness of the different taxonomic classifiers on HUMAnN 3 functional analysis, the taxonomic profiles generated by MetaPhlAn 4, mOTUs3, Centrifuge, and Kraken 2/Bracken were integrated into the HUMAnN 3 algorithm. The resulting functional profiles were then compared for each synthetic sample (Figure 5).

Functional analysis of the metatranscriptomes of synthetic samples performed using HUMAnN 3 in combination with the taxonomic classifiers MetaPhlAn 4, mOTUs3, Centrifuge, and Kraken 2/Bracken. Upper lollipop plots showing the number of gene families identified in each synthetic sample by HUMAnN 3 combined with different classifiers. Middle bar plots showing the abundance of mapped and unmapped functions as copies per million (CPM) in each synthetic sample. Unmapped represents the reads that failed to map after both HUMAnN 3 alignment steps (nucleotide and translated searches). Bottom heat maps showing the contribution of each search tier for functional analysis as CPM in each synthetic sample.

Overall, there was a gradual reduction in the number of functional features identified by HUMAnN 3 with the increase in host content of samples (Pearson’s r −0.98 to −0.99, *p* < .005; Supplementary Figure S3). In the Mock and SS10, a high number of gene families was identified and there was a low abundance of reads that failed to map after both nucleotide and translated searches (unmapped). The analysis of each search tier, showed that the functional features of Mock and SS10, were mostly explained by the nucleotide search, with a low abundance of functions coming from the translated search (143 to 802 CPM; Figure 5). In contrast, in SS70, SS90, and SS97, the number of functional features identified by HUMAnN 3 was lower, with an increased proportion of unmapped reads compared with Mock and SS10. Moreover, along with the increase in host content, there was a decline in the contribution of the nucleotide search and an increase in that of the translated search (Figure 5 and Supplementary Figure S3).

There was a major influence of the taxonomic classifier on the functional profiles of samples with lower microbial biomass (SS70, SS90, and SS97), which was not observed for samples with high microbial load (Mock and SS10; Figure 5). In SS70, SS90 and SS97, the use of marker- and *k*-mer- based methods resulted in distinct HUMAnN 3 functional profiles, with a reduction in the number of functions and an increase of unmapped reads in marker-based methods. These differences were even more pronounced in SS97. In this sample, the use of the *k*-merbased approaches resulted in a higher abundance of functions identified in the nucleotide search, demonstrating the importance of the taxonomic profile input.

Combining results from the taxonomic and functional analyses (Figures 2 & 5), the use of optimized Kraken 2/Bracken had the best F1-score and led to HUMAnN 3 identification of the highest number of microbial functions and to the lowest proportion of unmapped reads in the metatranscriptome of SS97.

We next compared the performance of HUMAnN 3 combined with the optimized Kraken 2/Bracken with that of the SAMSA2 pipeline (Supplementary Figure S4). We selected SAMSA2 because it follows HUMAnN 3 strategy of quantifying microbial functions from shotgun sequencing reads (mapping-based approach) rather than from assembled contigs.^22^ SAMSA2 had a similar performance to that of optimized Kraken 2/Bracken in identifying bacterial species of the mock community. However, the microbial profiles of SAMSA2 had a very high number (from 540 to 6015 species) of false-positives (Supplementary Figure S4A). Furthermore, SAMSA2 found an overall lower number of microbial functions (Supplementary Figure S4B), being outperformed overall by HUMAnN 3 combined with the optimized Kraken 2/Bracken.

Taken together, our results demonstrate the effectiveness of HUMAnN 3 for metatranscriptomics analysis in samples with low ratio of microbial to host cells, when combined with Kraken 2/Bracken.

### Effective application of the metatranscriptomics strategy in human gastric tissues

To test the applicability of our metatranscriptomics method in human clinical samples where the load of bacteria is considerably lower than the host content, we profiled five gastric tissue specimens with the same experimental and computational workflows used for synthetic samples.

The metatranscriptome datasets of the clinical tissue specimens produced a large number of raw single-end reads, ranging from 102 to 126 million. As expected, due to the high proportion of host cells in these samples, after sequence pre-processing, the number of reads considerably decreased to a mean of 168,695 reads per sample (Supplementary Table S6). This is comparable to the low number of reads of the SS97 dataset (198,545 reads; Supplementary Table S2).

**Figure 6.**
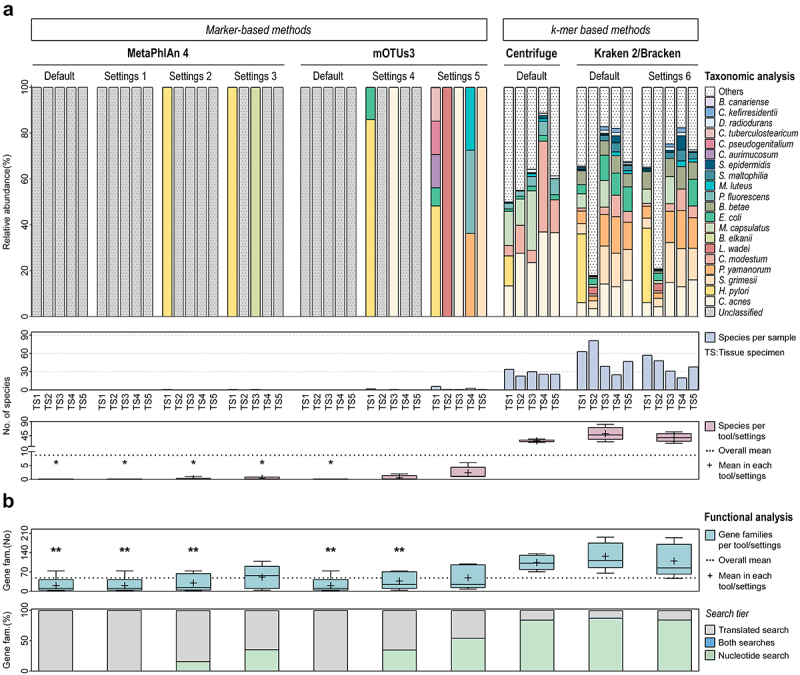
Effective application of the metatranscriptomics method in human clinical samples.

Major differences were observed between the taxonomic profiles of marker- and *k*-mer- based methods, despite the intrinsic variability of the clinical samples (Figure 6a).

(a) Taxonomic analysis at the species-level of the metatranscriptomes of clinical tissue specimens (TS) using MetaPhlan 4, mOTUs3, Centrifuge, and Kraken 2/Bracken with different parameter settings. Upper color-coded bar plot showing the relative abundances of the top 20 most abundant species in tissue specimens identified by each classifier. Others represent other identified species not shown in the graph (not in the top 20). Unclassified stands for no taxa identified. The number of species each tool/settings was able to identify is shown per tissue specimen (middle bar plot) and as median of all tissue specimens (bottom box plot). Significance was obtained using the Kruskal-Wallis non-parametric test, corrected with Dunn’s test for multiple comparisons. (b) Functional analysis of the metatranscriptomes of clinical tissue specimens using HUMAnN 3 in combination with MetaPhlan 4, mOTUs3, Centrifuge, and Kraken 2/Bracken with different parameter settings. Box plot showing the number of gene families that HUMAnN 3 was able to detect in tissue specimens using each tool/settings. Significance was obtained using the one-way ANOVA followed by Dunnett’s multiple comparisons test. Color-coded bar plot showing the contribution of each search tier for functional analysis of tissue specimens, represented as the proportion of gene families. * stands for significantly different from Kraken 2/Bracken (Settings 6) at *p* < .05.

MetaPhlAn 4 and mOTUs3 were the less sensitive classifiers to profile the metatranscriptome of human tissue specimens, consistent with their lowest recall values in SS97 (Figure 2). Strikingly, MetaPhlAn 4 and mOTUs3 with default parameters did not identify any species in any tissue specimen. Changing the settings of these classifiers, only slightly increased the number of detected species. In contrast, *k*-mer based methods markedly improved the identification of species in clinical tissue specimens, with Centrifuge and Kraken 2/Bracken having a mean number of detected species above the overall mean of all classifiers (Figure 6a). Reinforcing this aspect, the mean number of species in the taxonomic profiles of optimized Kraken 2/Bracken (settings 6) was significantly higher than in those of marker-based methods. A similar trend was observed in comparison with Centrifuge, but without statistical significance (Figure 6a).

These results confirm our previous findings in synthetic samples and demonstrate that Kraken 2/Bracken outperforms the other classifiers in characterizing the metatranscriptome of human tissues with low ratio of microbial to host cells.

By examining the functional profiles of tissue specimens generated by HUMAnN 3 in combination with different taxonomic classifiers, we observed a higher number of functions identified with *k*-mer *vs* marker-based methods (Figure 6b). Functional analysis using Centrifuge and Kraken 2/Bracken resulted in a mean number of gene families above the overall mean. In particular, these values were significantly higher with optimized Kraken 2/Bracken than those obtained with MetaPhlAn 4 and mOTUs3. When Kraken 2/Bracken profiles were used, 86% of the gene families were identified by the nucleotide search (the highest among all classifiers). In contrast, when profiles of marker-based methods were used, the low number of detected functions were identified by translated search (Figure 6b). These results show that the use of Kraken 2/Bracken highly improved HUMAnN 3 functional analysis of clinical tissue specimens, by increasing the number of gene families identified in comparison with marker-based methods.

Overall, we demonstrated the value of the metatranscriptomics workflow to characterize the microbiome of human tissues of the GI tract with low microbial content.

## Discussion

Metatranscriptome analysis of human tissues with low microbial content remains challenging due to the low ratio of microbial to host cells, and to the absence of an optimized bioinformatics workflow specifically tailored to profile this type of samples. Here, we propose a strategy that integrates the taxonomic analysis of Kraken 2/Bracken with the functional analysis of HUMAnN 3 to achieve maximum resolution in the characterization of the metatranscriptome of samples with low bacterial concentrations.

The *k*-mer based methods proved to be the best-performing tools for taxonomic classification of the metatranscriptome of samples with low ratio of microbial to host cells, due to their high sensitivity. Specifically, Kraken 2/Bracken with optimized settings maximized the accuracy of species detection by identifying a higher number of bacteria, while minimizing false-positives. These results are in accordance with those of previous metagenomics studies showing that this *k*-mer based method was among the most accurate taxonomic classifiers.^23,24^ Our results are also consistent with a recent report showing that optimizing the confidence threshold parameter within Kraken 2 is fundamental to improve precision.^25^ Our data show that marker-based methods are less effective at profiling the samples with the lowest microbial biomass than *k*-mer based methods, due to their decreased sensitivity as the host content of samples increases. In line with these observations, a study benchmarking metagenomics tools showed that MetaPhlAn 2 and mOTUs2 had lower recall compared with Kraken 2 and Bracken.^24^ The two types of classification tools rely on distinct algorithms and databases, which are likely to contribute to the performance differences observed. While *k*-mer based methods classify reads against a database of whole genomes, marker-based methods use a database that only includes a subset of gene sequences, namely species-specific phylogenetic markers.^21,26^ Still, our results show that marker-based methods have good performance in profiling the metatranscriptome of samples with high microbial load. In fact, MetaPhlAn has been widely used in metatranscriptomics studies of stool samples.^2,5,20,27^ In this scenario, the high abundance of microbial sequences allows an easy identification of taxa using a database of phylogenetic markers. In contrast, in samples where the ratio of microbial to host cells is low, we hypothesize that the number of microbial sequences is insufficient for the detection of phylogenetic markers, because these tools only identify a few genes per genome, using a small portion of the total sequencing depth. Taken together, our data emphasize both high sequencing depth and the use of sensitive taxonomic classifiers as essential requirements for an efficient characterization of tissue samples with low microbial load. In addition, the use of a comprehensive and most updated database, including rare or recently discovered species, is recommended to avoid inaccurate classifications.

One of the main goals of metatranscriptomics is to measure the functional activity of the microbiome.^10^ However, there are few mapping-based methods validated for functional profiling of metatranscriptomics data.^21,22,28^ Here, we used one such method, HUMAnN 3, which applies a tiered search to identify microbial functions and to stratify them by species’ contributions.^21^ Using HUMAnN 3, we detected a higher number of functional features in the metatranscriptome of samples with high vs. low microbial content, which is consistent with its successful use in metatranscriptomics of stool specimens.^5,20,29^ Our data also revealed major differences in the functional profiles when using marker- and *k*-mer- based methods in samples with the lower microbial biomass. The latter, and in particular Kraken 2/Bracken, improved functional analysis by identifying a large number of gene families mainly using the nucleotide search. This highlights the importance of the taxonomic profile for HUMAnN 3 functional analysis of this type of samples. It also suggests that in samples with low ratio of microbial to host cells, it might be reasonable to bypass the computationally intensive translated search, when a sensitive taxonomic classifier is used. Furthermore, the combined strategy of optimized Kraken 2/Bracken integrated with HUMAnN 3 outperformed the SAMSA2 pipeline, detecting fewer false positives, while identifying a higher number of functions in synthetic samples.

The use of human tissues of the GI tract demonstrated proof of concept for the applicability of the metatranscriptomics strategy in clinical samples with low microbial content. The successful improvement in the resolution of the taxonomic and functional features of the mucosal microbiome, which was a limitation of previous computational workflows, will benefit downstream analyses. For example, the use of more accurate microbial profiles will generate improved co-expression gene networks, differential gene expression, and functional enrichment analyses in host-microbiome interaction studies.

In conclusion, this study provides valuable guidance for future design of metatranscriptomic assays in the challenging setting of clinical tissue samples, highlighting the importance of using microbiome standards to establish robust computational workflows and selecting appropriate bioinformatics tools based on research goals and sample types. An accurate characterization of the metabolically active members of the mucosal microbiome will improve our understanding of their dynamics and their functional interactions with the host, in the context of health and disease.

## Materials and methods

### Mock microbial community

The mock microbial community “20 Strain Even Mix Whole Cell Material” (ATCC® MSA-2002™) was developed by American Type Culture Collection (ATCC) as a microbiome reference standard. This mock microbial community is a high complex mixture of 20 bacterial species, prepared as lyophilized whole cells (3×10^7^ cells/vial) at an even number of cells (1.5×10^6^ cells/species). It comprises fully sequenced and characterized strains selected on the basis of relevant phenotypic and genotypic features, such as Gram stain, GC content, and genome size (Supplementary Table S1). The reconstitution of the lyophilized bacterial mixture was performed by gently resuspending the pellet in 1 mL of cold PBS. The bacterial cell density was inferred from the total number of lyophilized cells in the mock vial specified by the manufacturer.

### Cell culture

The AGS cell line (human gastric adenocarcinoma cell line, ATCC CRL-1739) was cultured in RPMI-1640 medium (with GlutaMAX and HEPES) (Gibco, Invitrogen, CA, USA) supplemented with 10% fetal bovine serum (HyClone, Perbio) and 1% penicillin/streptomycin (Gibco, Invitrogen). The cells were incubated at 37°C under 5% CO_2_ humidified air. The human cell line density was measured using the TC20 automated cell counter (Bio-Rad, California, USA).

### Generation of synthetic host-microbiome samples and RNA isolation

To create synthetic samples (SS) mimicking human samples with different microbial loads, the whole cell mock microbial community of well-defined bacterial composition (ATCC MSA-2002) was spiked with the human cell line (AGS, ATCC CRL-1739). SS with increasing ratios of host to bacterial cells were prepared containing 10% (SS10), 70% (SS70), 90% (SS90), and 97% (SS97) host cells.

Each synthetic sample comprised a total of 1 × 10^7^ cells (bacteria and human). The exact volumes of bacterial and human cells suspension to be mixed in each condition were calculated according to the mock community and human cell line densities.

RNA isolation from synthetic samples, mock microbial community (Mock), and human cell line (AGS) was immediately performed using the AllPrep® Bacterial DNA/RNA/Protein Kit (Qiagen, Hilden, Germany) according to the manufacturer’s instructions. Initially, bacterial cells were first re-suspended in a chaotropic formulation (Solution HC containing β-mercaptoethanol (β-ME)), followed by a mechanical homogenization using 0.1 mm glass beads for optimal lysis of both gram-positive and gram-negative bacteria and for solubilization of total bacterial nucleic acids. The resulting lysate was combined with a solution to selectively capture the RNA on a silica-based membrane. Finally, the immobilized RNA was washed, and eluted in Microbial DNA-free/RNase-free water (Qiagen). To avoid DNA contamination, after RNA isolation, samples were treated with DNase I (2 U/µL; New England BioLabs Inc., MA, United States), followed by a cleanup step through column purification using the Monarch RNA Cleanup kit (New England BioLabs Inc).

### Gastric tissue specimens and RNA isolation

Gastric tissue specimens were collected from the Tissue and Tumor Bank of the Centro Hospitalar Universitário S. João (CHUSJ), which follows the principles on data protection and informed consent established in the Portuguese law (Lei nº 12/2005, de 26 de Janeiro). The use of these samples has been approved by the Ethics Committee of CHUSJ (Project 441/19). RNA was isolated from the tissue specimens using the AllPrep® DNA/RNA Mini kit (Qiagen) following the manufacturer’s instructions. Initially, a fragment of each tissue specimen embedded in optimal cutting temperature compound (OCT) was cut and the OCT removed. To avoid cross-contamination, a single sterile bistoury was used per sample. Frozen tissue specimens were first disrupted and homogenized in Buffer RTL Plus (contains β-mercaptoethanol) using the TissueRuptor (Qiagen). Then, the lysate was added to the AllPrep DNA spin column, and the resulting flow-through was combined with ethanol (70%), before being transferred onto the RNeasy spin column. DNase I (Grisp, Porto, Portugal) digestion was performed on the RNeasy spin column membrane to remove potential DNA contaminations. Finally, RNA bounded in the column membrane was washed successively (to eliminate contaminants and DNase I), and eluted in pre-heated Microbial DNA‐free/RNase-free water (Qiagen). The OCT without tissue was used as control, and was processed for RNA isolation using the same protocol.

### RNA quantity, purity and integrity

The RNA quantity, purity, and integrity were assessed using the Qubit® 3.0 Fluorometer (ThermoFisher Scientific, Massachusetts, USA) (Supplementary Table S7), the Nanodrop^TM^ One UV spectrophotometer (ThermoFisher Scientific), and the Agilent Bioanalyzer 2100 (Agilent Technologies, Palo Alto, California), respectively.

### Library preparation and metatranscriptome sequencing

The sequencing library was prepared using the TruSeq Stranded Total RNA kit (Illumina Inc., CA, United States) according to the manufacturer’s protocol. Briefly, to enrich the amount of mRNA, a double depletion of prokaryotic (NEBNext rRNA Depletion Kit (Bacteria), New England BioLabs, Cat. No. NEB #E7850) and eukaryotic (cytoplasmic and mitochondrial; Ribo-Zero Gold kit, Illumina Inc, Cat. No. RS-122-2301) rRNAs was applied. Following this, the RNA was purified and randomly fragmented using divalent cations under high temperature. The cleaved RNA fragments were reverse transcribed into first strand complementary DNA (cDNA) using reverse transcriptase and random primers, followed by second strand cDNA synthesis using DNA Polymerase I and RNase H. After addition of a single “A” nucleotide to the 3’ ends of the cDNA and subsequent ligation of the indexing adapters for hybridization onto the flow cell, a limited-cycle PCR was performed to enrich for these cDNA fragments, followed by a cleanup step that purified the library, removing small fragments by using Agencourt AMPure XP beads (Beckman Coulter, Inc., CA, United States). The library quality and quantity were assessed using the Agilent Technology 2100 Bioanalyzer (Agilent Technologies) and qPCR, respectively. Library sequencing was performed as a paired-end 150-cycle run on the Illumina NovaSeq 6000 platform with a depth of 15 Gbp per sample. The raw sequencing data has been deposited at the NCBI Sequence Read Archive (PRJNA1003801).

### Metatranscriptome sequencing data analysis

#### Sequencing data pre-processing

Raw metatranscriptomic reads were pre-processed with KneadData (version 0.10.0),^21^ which uses the tools FastQC (version 0.11.9),^30^ Trimmomatic (version 0.33),^31^ and Bowtie 2 (version 2.4.4),^32^ to do quality check, quality filtering, and human, viruses, and rRNA sequences removal, respectively.

Initially, reads were trimmed based on a sliding window trimming approach, cutting once the average base Phred quality score within a 4-base sliding window was < 20. When more than half of the read length was trimmed, the reads were discarded. Illumina adapters and repetitive/overrepresented sequences were also removed during quality filtering (Trimmomatic options --sequencer-source and --run-trim-repetitive). Further, KneadData used Bowtie 2 to identify and remove human, viruses, and rRNA reads. Human sequences were filtered out by removing reads that aligned to the human genome (hg37dec_v0.1) and human transcriptome (human_hg38_refMrna) references.^21^ Viruses and rRNA sequences were additionally depleted from the datasets by mapping the reads against a custom database of all RefSeq complete viral genomes (downloaded from NCBI Virus in November 2021) and the SILVA 128 LSU and SSU Parc ribosomal RNA reference database,^33^ respectively.

#### Taxonomic profiling

Quality-controlled reads were taxonomically classified using different computational methods. A description of all classifiers used, and parameter settings applied is listed in Table 1.

##### MetaPhlAn 4 (version 4.0.0)

MetaPhlAn 4 is an assembly-free profiler that maps the quality-controlled shotgun reads to a database of unique clade-specific nucleotide markers using Bowtie 2, to estimate the set of microbial species present in samples and their relative abundances.^34,35^ MetaPhlAn 4 expands existing capabilities of MetaPhlAn 3 by incorporating a new markers database that integrates information from both reference and metagenomic assembled genomes.^35^ The database used was the default (mpa_vJan21_CHOCOPhlAnSGB_202103), which contains ~ 5.1 million unique clade-specific marker genes identified from ~ 1 million microbial genomes (~236,600 microbial isolates and 771,500 metagenomic assembled genomes) for 26,970 species-level genome bins.^35^ Internally, MetaPhlAn 4 estimates the relative abundance of each clade by calculating the coverage of each clade as the robust average of the coverage of all markers of the same clade, and then, by normalizing the clade’s coverage across all detected clades. In order to improve the taxonomic classification, several settings were used by altering the --stat_q and/or the --min_mapq_val parameters (Table 1). The --stat_q parameter represents the quantile value for the robust average, excluding the top and bottom quantiles of the marker abundances. To include more markers in the analysis, MetaPhlAn 4 was run with lower --stat_q values than the default. The --min_mapq_val parameter is the minimum mapping quality value (MAPQ). In order to use all the alignments, ignoring the mapping quality, the --min_mapq_val was set to −1.

##### mOTUs3 (version 3.0.1)

mOTUs3 is a software tool that identifies species and quantifies their relative abundance by assigning reads to a sequence database of universal, protein coding, single-copy phylogenetic marker genes.^36,37^ These markers were extracted from ~ 86,000 prokaryotic reference genomes, more than 3,100 metagenomic samples, and ~ 600,000 metagenome assembled genomes. Clusters of marker genes at the species-level led to the generation of a database of marker genes-based operational taxonomic units (mOTUs) containing 11,915 reference mOTUs (ref-mOTUs), 2,297 metagenomic mOTUs (meta-mOTUs), and 19,358 metagenome assembled genomes mOTUs (ext-mOTUs).^37^ The database used in the analysis was the db_mOTU_v3.1.0. The relative abundance of each mOTUs is calculated as the median across all its respective marker gene clusters abundances with non-zero values. For that, we used the default option -y insert.scaled_counts, which normalizes the number of mapped reads by marker gene length and then rescales by total abundance. To improve species identification, several settings were used by altering the -g parameter (Table 1). The -g parameter controls the minimum number of detected marker gene clusters required to call a species as being present in a sample (detection threshold for a mOTUs). To improve sensitivity, mOTUs3 was run with lower values for the -g option than the default.

##### Centrifuge (version 1.0.4)

Centrifuge is a microbial classification tool using an indexing scheme based on the Ferragina-Manzini index and Burrows-Wheeler transform, which allows the construction of significantly smaller databases and the classification of reads at high speed with low memory requirements.^38^ This classifier relies on exact *k*-mer matching, assigning a read to multiple taxonomic categories. Besides reads classification, Centrifuge also performs taxa abundance estimation at different taxonomic ranks through an Expectation-Maximization algorithm. The database we used for the analysis was the p.compressed (15 April 2018 version) containing complete bacterial and archaeal RefSeq genomes compressed. Centrifuge was run using the default classification options that include the --min-hitlen 22 and -k 5 (Table 1). The --min-hitlen represents the minimum length of the assignments to classify a read. The -k option is the maximum number of taxonomic labels per sequence.

##### Kraken 2 (version 2.1.2) and Bracken (version 2.6.2)

Quality-controlled reads were subjected to taxonomic classification using Kraken 2.^26,39^ The classification algorithm of Kraken 2 uses exact alignment of read *k*-mers (sub-sequences of length *k*) against a database of *k*-mers from existing genomes. Each *k*-mer within a read is labeled with the lowest common ancestor taxa containing the given *k*-mer. The Kraken 2 database used for the analysis was the Standard-16 (17 May 2021). This database is the standard database of Kraken 2 and contains complete genomes from RefSeq for Bacteria, Archaea, Viruses, along with the GRCh38 assembly of the human genome, and a collection of known vectors (UniVec_Core). In order to reduce the false-positives detected, improving the classification accuracy, a different setting was used by altering the --confidence option in Kraken 2 (Table 1). The confidence score threshold is the minimum proportion of *k*-mers mapping to a taxon needed to take that taxonomic classification. When the confidence score given to a sequence is below this threshold, the sequence is called unclassified by Kraken 2.

The relative abundance of species within a sample was then estimated using Bracken (Bayesian Reestimation of Abundance after Classification with Kraken).^40^ This is a statistical approach that computes the relative abundance of species by estimating the number of reads originating from each species using the taxonomic assignments of Kraken 2. This method probabilistically reassigns reads in the taxonomic tree from intermediate taxonomic nodes to the species level.

#### Decontamination of the taxonomic profile

Decontam^41^ is an R package that provides statistical methods to identify contaminants in sequencing data. The decontam function “*isContaminant”* was used with the frequency mode, which based the identification of contaminants on the distribution of taxa frequencies as a function of samples concentration (determined by qPCR during library preparation). When the score statistic of a sequence feature is lower than the threshold, the sequence feature is classified as a contaminant. Putative lists of microbial contaminants per sample were then created and these taxa were removed from the taxonomic profiles. To assure that taxa truly present in a sample were not incorrectly removed by decontam, a set of microorganisms that could not be discarded were defined. For synthetic samples, it comprised the 20 bacterial species from the mock community, and for gastric tissue specimens, included species *Helicobacter pylori*, a bacterium that colonizes the gastric epithelium. Additionally, species identified by the NCBI Taxonomy database as unclassified taxa (reflecting either ambiguity of assignment in a specific rank or represent poorly defined names in the NCBI classification) were also removed from the taxonomic profiles. After discarding contaminant and unclassified species, the relative abundances of the remaining taxa were re-calculated.

#### Metrics (precision, recall, and F1 score)

The metrics precision, recall and F1 score were used to measure the performance of classifiers, evaluating how well they detect the presence or absence of species of the mock community regardless of their relative abundances. Precision and recall were calculated based on the number of species of the mock community, where precision was the proportion of true positive species identified in the sample (mock species detected) divided by the number of total species identified by the classifier (mock species + others), and recall was the proportion of true positive species (mock species detected) divided by the number of different species actually in the sample (total number of mock species). The F1 score is the harmonic mean of precision and recall, giving equal weight to them in a single metric (2 × (precision x recall/precision + recall)).

#### Functional profiling

##### HUMAnN 3 (version 3.6)

HUMAnN 3 (HMP Unified Metabolic Analysis Network) is a method that performs functional analysis to determine the metabolic potential of the members of a microbial community.^21^ This approach identifies and quantifies microbial functions from metagenomics and metatranscriptomics data. Initially, a taxonomic pre-screening that identifies the microbial species present in each sample is performed. Based on this taxonomic profile, HUMAnN 3 selects pangenomes from the full ChocoPhlAn database to create a sample-specific reference database, containing only the pangenomes of the subset of species detected in the sample. By default, this pre-screen is performed by MetaPhlAn, but a custom taxonomic profile can also be supplied to HUMAnN 3. Quality-controlled reads are then mapped against this sample-specific pangenome database using bowtie 2 to quantify genes presence and abundance per-species (Nucleotide search). For all the reads that fail to map at the nucleotide search, a translated alignment is performed against the comprehensive and non-redundant UniRef-based protein database using DIAMOND (Translated search). This tiered search yields functional profiles, in which the gene families identified and quantified are stratified by each species contributing to those functions. Gene family abundance is reported by default in reads per kilobase (RPK) units, which were then sum-normalized to copies per million (CPM) units to account for differences in sequencing depth. The databases used for the analysis were the full ChocoPhlAn pangenome database (v3.1, January 2019) and the UniRef90 EC filtered database (version 20191b). To improve nucleotide search, maximizing the number of mapped reads, the coverage threshold was set to 25, using the option --nucleotide-subject-coverage-threshold.

The decontaminated taxonomic profiles from all classifiers (MetaPhlAn 4, mOTUs3, Centrifuge, and Kraken 2/Bracken) were used as input for the taxonomic pre-screening step. Taxa with very low abundance were removed from the analysis using the --prescreen-threshold option. This parameter was set to 0.001, to exclude taxa with an abundance lower than 0.001.

##### SAMSA2 (version 2.2.0).

The Simple Annotation of Metatranscriptomes by Sequencing Analysis 2 (SAMSA2) is an entire standalone bioinformatics pipeline that evaluates changes in transcript abundances at the community level.^22^ The SAMSA2 analysis pipeline starts with a pre-processing step, in which sequencing reads are merged, low-quality reads and/or adaptor sequences are removed, and rRNA sequences are filtered out. Following this, mRNA is the annotated using DIAMOND against a selected reference database. The annotated results are condensed and converted into sorted abundances counts by functions and organisms.

The pre-processing step performed by SAMSA2 was bypassed in our analysis, since quality-controlled metatranscriptomes were generated using KneadData. The database used for organism and functional annotation was created from the NCBI RefSeq database (complete_nonredundant_prt).

### Generation of simulated datasets of synthetic host-microbiome samples with high host/bacteria ratios

Simulated datasets (SD) of the metatranscriptomes of synthetic samples with low microbial biomass and progressively greater proportions of host reads were constructed, using an in-house script. Microbial and host reads were randomly selected from our sequenced datasets using seqtk (v1.3-r117-dirty) at a fixed sequencing depth of 100 million reads (15 Gbp). Microbial single-end reads were randomly picked from the pre-processed Mock dataset, to guarantee that only microbial reads were selected, while host single-end reads were randomly selected from the human sequences discarded by KneadData from the SS97 dataset, to assure sufficient host sequences. Five simulated datasets were created containing 90%, 97%, 98%, 99%, and 100% host reads (SD90, SD97, SD98, SD99, and SD100, respectively). Three independent experiments were performed using a random seed for each experiment (Seeds for experiment 1, 2, and 3: 262, 988, and 774, respectively).

### 16S rRNA transcript sequencing

RNA samples were reverse transcribed using the SuperScript IV First-Strand kit (ThermoFisher Scientific). The 16S rRNA transcript was amplified from the cDNA samples using universal primers fused with Illumina adapters sequences U789F_v56_ngs 5′‐ TCGTCGGCAGCGTCAGATGTGTATAAGAGACAGTAGATACCCBDGTAGTCC‐3′ and U1053R_v56_ngs 5′- GTCTCGTGGGCTCGGAGATGTGTATAAGAGACAGCTGACGACRRCCATGC‐3′ (Integrated DNA Technologies) targeting the V5-V6 hypervariable regions. The PCR reactions were performed in 35 μL containing 1× AmpliTaq Gold 360 Master Mix (Applied Biosystems, Foster City, CA, USA) and 0.4 μM of forward and reverse primers (Integrated DNA Technologies). Microbial DNA‐free water (Qiagen) was added to PCR negative controls instead of cDNA. PCR was performed with 9 min of pre-denaturation at 95°C, followed by 33 cycles of 30 seconds at 95°C, 45 seconds at 52°C, and 45 seconds at 72°C. Final extension was performed for 10 minutes at 72°C. Amplicons were subsequently purified with magnetic beads using the Axy Prep PCR Clean‐Up Kit (Axygen, Union City, CA, USA), visualized in 1.5% agarose gels and quantified with the Qubit dsDNA HS Assay Kit (Life Technologies, Foster City, CA, USA). Equal concentrations of amplicons were used for library preparation to incorporate barcode sequences. Sequencing was performed on the Illumina MiSeq platform, as a paired-end 150-cycle run, with an expected output of 100,000 reads per sample. Negative cDNA synthesis and RNA extraction controls were also sequenced to monitor contaminations during the entire workflow.

### 16S rRNA transcript sequencing data analysis

Raw paired‐end reads were assembled, and primer sequences were removed at both ends. Reads were quality filtered by imposing a maximum number of expected errors of 1.0, and trimmed to a fixed length. Filtered reads were dereplicated and denoised to produce amplicon sequence variants (ASVs) using the UNOISE algorithm.^42,43^ The minimum number of reads for an ASV to be considered was set to 8. ASVs were aligned against the 18S rRNA (SILVA 18S rRNA v123) and the internal transcribed spacer (RDP ITS v2) sequences to identify and remove putative eukaryotic contaminations. An ASVs table was constructed by mapping raw reads back to ASVs to get counts per sample. Further, each biological sequence was taxonomically assigned with SINTAX algorithm^44^ using the 16S RDP Classifier training set v16 as the reference database and with a confidence threshold of 0.4. Putative bacterial contaminants were flagged and removed using decontam,^41^ if they were identified in increase prevalence in negative controls or their frequency varies inversely with samples concentration. Sequencing data analysis was performed using usearch_v11.0.667_i86linux32.

### Statistical analyses

The GraphPad Prism software (version 8.02, San Diego, CA, USA) was used to perform the statistical treatment. D’Agostino-Pearson omnibus K2 and Shapiro‐Wilk normality tests were applied to evaluate whether the data followed a normal distribution. Pearson’s and Spearman’s correlation were used to measure correlations between the percentage of host cells in synthetic samples and multiple variables in the taxonomic and functional analyses. A Spearman’s correlation matrix was used to measure the correlation between the taxonomic profiles from 16S rRNA transcript sequencing and metatranscriptomics with Kraken 2/Bracken. Comparisons between groups, when performing the simulated datasets analysis, and the taxonomic and functional analyses in gastric tissue specimens, were performed using the one-way analysis of variance (ANOVA) followed by Dunnett’s multiple comparisons test and the Kruskal-Wallis non-parametric test, corrected with Dunn’s test for multiple comparisons. The differences were considered statistically significant with *p* values lower than 0.05.

## Supplementary Material

Supplemental Material

## Data Availability

The raw sequence data of the datasets generated in the current study are available at the NCBI BioProject under the accession number: PRJNA1003801.
