# Supplementary material for: A metatranscriptomics strategy for efficient characterization of the microbiome in human tissues with low microbial biomass
Source: Gut Microbes. 2024 Feb 29;16(1):2323235. doi: 10.1080/19490976.2024.2323235 (PMC10913719; doi:10.1080/19490976.2024.2323235)
Supplement: Supplemental Material [file KGMI_A_2323235_SM1526.pdf]

SUPPLEMENTAL MATERIAL

A metatranscriptomics strategy for efficient characterization of the microbiome in human tissues with low microbial biomass

Joana Pereira-Marques, PhD [a,b], Rui M. Ferreira, PhD [a,b,*], Ceu Figueiredo, PhD [a,b,c,*]

[a]i3S – Instituto de Investigação e Inovação em Saúde, Universidade do Porto, Rua Alfredo Allen 208, 4200-135 Porto, Portugal;

[b]Ipatimup – Institute of Molecular Pathology and Immunology of the University of Porto, Rua Júlio Amaral de Carvalho 45, 4200-135 Porto, Portugal;

[c]Department of Pathology, Faculty of Medicine of the University of Porto, Alameda Prof. Hernâni Monteiro, 4200 – 319 Porto, Portugal.

[*]These authors contributed equally.

Corresponding Author: Ceu Figueiredo, Phone: +351 225570700; e-mail: cfigueiredo@i3s.up.pt

SUPPLEMENTARY TABLES

Supplementary Table S1. Composition of the mock microbial community (ATCC® MSA-2002™), showing relevant phenotypic and genotypic features, such as Gram staining, GC content, genome size, and 16S rRNA copies per genome.

Species name	ATCC number	Gram staining	GC content (%) #	Genome size (Mb) #	16S rRNA copies per genome #
Acinetobacter baumannii	17978™	Negative	38.9	4.07	6
Bacillus pacificus	10987™	Positive	35.5	5.44	14
Bifidobacterium adolescentis	15703™	Positive	59.2	2.08	5
Cereibacter sphaeroides	17029™	Negative	69.0	4.48	4
Clostridium beijerinckii	35702™	Positive	29.9	6.00	15
Cutibacterium acnes	11828™	Positive	60.0	2.49	3
Deinococcus radiodurans	BAA-816™	Positive	66.7	3.28	3
Enterococcus faecalis	47077™	Positive	37.8	2.73	4
Escherichia coli	700926™	Negative	50.8	4.64	7
Helicobacter pylori	700392™	Negative	38.9	1.66	2
Lactobacillus gasseri	33323™	Positive	35.2	1.85	6
Neisseria meningitidis	BAA-335™	Negative	51.5	2.24	4
Phocaeicola vulgatus	8482™	Negative	42.2	5.16	7
Porphyromonas gingivalis	33277™	Negative	48.3	2.39	4
Pseudomonas paraeruginosa	9027™	Negative	66.6	6.37	4
Schaalia odontolytica	17982™	Positive	65.4	2.39	3
Staphylococcus aureus	BAA-1556™	Positive	32.7	2.92	6
Staphylococcus epidermidis	12228™	Positive	32.1	2.57	6
Streptococcus agalactiae	BAA-611™	Positive	35.6	2.16	7
Streptococcus mutans	700610™	Positive	36.8	2.03	5

ATCC Genome Portal (https://genomes.atcc.org/)

Supplementary Table S2. Summary of the sequencing data pre-processing of the metatranscriptomes from synthetic host-microbiome samples (SS).

Sample	Total no. of raw single-end reads	Total no. of quality-filtered reads	(%)	Total no. of quality-controlled reads	(%)
Mock	102,610,026	98,185,171	95.7	91,923,757	89.6
SS with 10% host cells	108,687,290	102,916,917	94.7	30,972,017	28.5
SS with 70% host cells	109,253,453	103,730,969	94.9	1,982,665	1.81
SS with 90% host cells	132,507,529	124,931,358	94.3	688,428	0.52
SS with 97% host cells	126,229,641	119,819,561	94.9	198,545	0.16

SS, synthetic sample.

Supplementary Table S3. Summary of the ribosomal, viruses, and human reads removed during sequencing data pre-processing of the metatranscriptomes from synthetic host-microbiome samples (SS).

Sample	No. of rRNA reads	No. of viruses reads	No. of human transcriptome reads	No. of human genome reads
Mock	3,917,525	1,814,163	1,699,275	2,213,820
SS with 10% host cells	6,741,686	10,610,128	59,057,878	65,276,146
SS with 70% host cells	6,859,452	12,703,469	85,062,293	93,802,613
SS with 90% host cells	7,708,973	13,486,131	106,351,370	116,254,914
SS with 97% host cells	7,109,878	13,577,218	103,962,473	112,048,031

SS, synthetic sample; rRNA, ribosomal RNA

Supplementary Table S4. Statistical analysis (*P* values) of the Spearman's correlation matrix between the taxonomic profiles of metatranscriptomics with optimized Kraken 2/Bracken and 16S rRNA transcript sequencing (Figure 3B).

***P* value**		*16S rRNA transcript sequencing*				
		**Mock**	**SS10**	**SS70**	**SS90**	**SS97**
Metatranscriptomics Kraken 2/Bracken	**Mock**	5.47E-05	1.15E-04	1.70E-04	3.20E-04	2.23E-04
	**SS10**	3.89E-06	4.68E-06	6.13E-06	2.07E-05	1.65E-05
	**SS70**	6.69E-06	6.13E-06	8.65E-06	1.65E-05	1.20E-05
	**SS90**	1.02E-05	1.02E-05	1.41E-05	2.77E-05	2.07E-05
	**SS97**	1.55E-07	9.97E-08	1.79E-07	1.79E-07	9.97E-08

SS, synthetic sample.

Supplementary Table S5. Statistical analysis (*P* values) of the mean relative abundances of species from simulated datasets (SD). For each simulated dataset, three replicates were randomly generated (Figure 4). The Kruskal-Wallis non-parametric test followed by multiple comparisons using Dunn's test was performed for each species.

Species name	SD90 vs. SD97	SD90 vs. SD98	SD90 vs. SD99	SD97 vs. SD98	SD97 vs. SD99	SD98 vs. SD99
Deinococcus radiodurans	>0.9999	>0.9999	>0.9999	>0.9999	>0.9999	>0.9999
Streptococcus mutans	>0.9999	0.1412	>0.9999	>0.9999	>0.9999	>0.9999
Pseudomonas paraeruginosa	>0.9999	>0.9999	>0.9999	>0.9999	>0.9999	>0.9999
Enterococcus faecalis	>0.9999	>0.9999	>0.9999	>0.9999	>0.9999	>0.9999
Cereibacter sphaeroides	>0.9999	>0.9999	>0.9999	>0.9999	>0.9999	>0.9999
Escherichia coli	0.3154	0.3154	0.1354	>0.9999	>0.9999	>0.9999
Streptococcus agalactiae	>0.9999	0.2492	>0.9999	>0.9999	>0.9999	>0.9999
Cutibacterium acnes	>0.9999	>0.9999	>0.9999	>0.9999	>0.9999	>0.9999
Acinetobacter baumannii	>0.9999	>0.9999	>0.9999	>0.9999	>0.9999	>0.9999
Bacillus pacificus	>0.9999	>0.9999	0.7343	>0.9999	>0.9999	>0.9999
Staphylococcus aureus	>0.9999	0.1887	0.2492	>0.9999	>0.9999	>0.9999
Porphyromonas gingivalis	>0.9999	>0.9999	>0.9999	>0.9999	>0.9999	>0.9999
Neisseria meningitidis	>0.9999	>0.9999	0.9331	>0.9999	>0.9999	>0.9999
Staphylococcus epidermidis	>0.9999	0.1887	0.4202	>0.9999	>0.9999	>0.9999
Schaalia odontolytica	0.354	>0.9999	0.4026	>0.9999	>0.9999	>0.9999
Bifidobacterium adolescentis	>0.9999	>0.9999	>0.9999	>0.9999	0.9485	>0.9999
Lactobacillus gasseri	>0.9999	>0.9999	>0.9999	>0.9999	>0.9999	>0.9999
Phocaeicola vulgatus	>0.9999	>0.9999	>0.9999	>0.9999	>0.9999	>0.9999
Clostridium beijerinckii	0.3378	0.4378	0.0656	>0.9999	>0.9999	>0.9999
Helicobacter pylori	>0.9999	>0.9999	>0.9999	>0.9999	>0.9999	>0.9999
Others	0.4144	0.2806	0.1191	>0.9999	>0.9999	>0.9999

SD, simulated dataset.

Supplementary Table S6. Summary of the sequencing data pre-processing of the metatranscriptomes from clinical tissue specimens (TS).

Sample	Total no. of raw single-end reads	Total no. of quality-filtered reads	(%)	Total no. of quality-controlled reads	(%)
TS1	126,108,056	111,866,898	88.7	596,550	0.47
TS2	109,866,673	100,077,081	91.1	124,573	0.11
TS3	124,262,941	113,788,766	91.6	48,097	0.04
TS4	108,374,169	96,214,794	88.8	31,813	0.03
TS5	102,252,565	95,606,397	93.5	42,444	0.04
Mean	114,172,881	103,510,787	90.7	168,695	0.14

TS, tissue specimen.

Supplementary Table S7. RNA quantification of synthetic host-microbiome samples (SS), and clinical tissue specimens (TS) using Qubit® 3.0 Fluorometer (ThermoFisher Scientific, Massachusetts, USA).

Sample	RNA concentration (ng/μL)
Mock	45.4
SS with 10% host cells	83.6
SS with 70% host cells	135.3
SS with 90% host cells	149.0
SS with 97% host cells	115.3
TS1	210.5
TS2	173.6
TS3	204.0
TS4	181.3
TS5	121.3

SS, synthetic sample; TS, tissue specimen.

SUPPLEMENTARY FIGURES

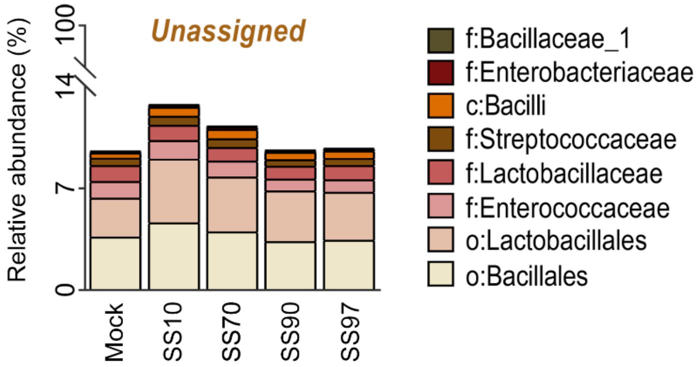

Supplementary Figure S1. Relative abundance of the unassigned taxa removed from the 16S rRNA transcript sequencing analysis of the synthetic host-microbiome samples (SS). ”Unassigned” comprises ASV’s that were not classified at the genus level. These taxa likely represent bacterial genera from the mock community that could only be identified at the family, order, and class level.

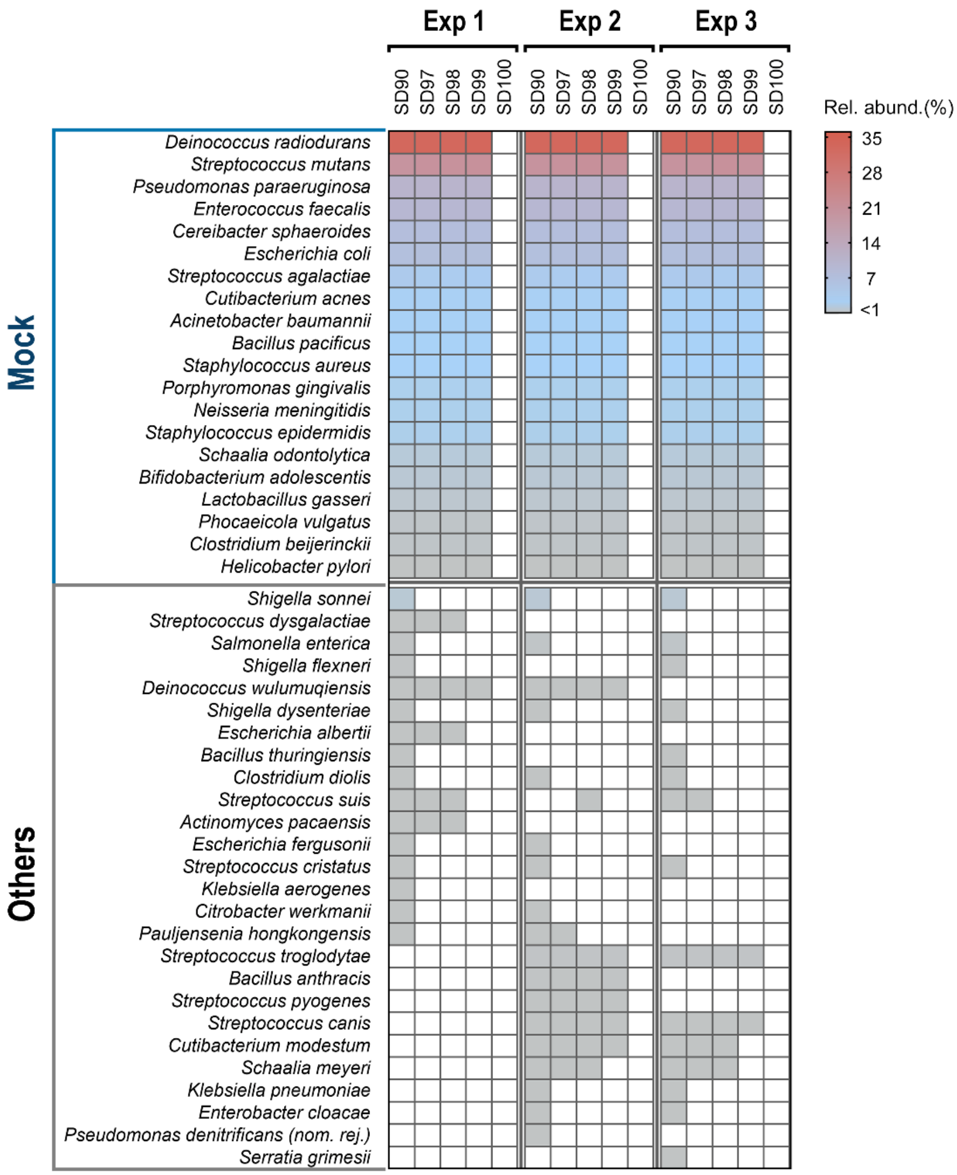

Supplementary Figure S2. Heat map showing the taxonomic profiles at the species-level of the five simulated datasets (SD) with progressively higher host sequences (90%, 97%, 98%, 99%, and 100%) determined using Kraken 2/Bracken with optimized settings, in each independent experiment. Others represent other microbial species identified that are not present in the mock community (false-positives).

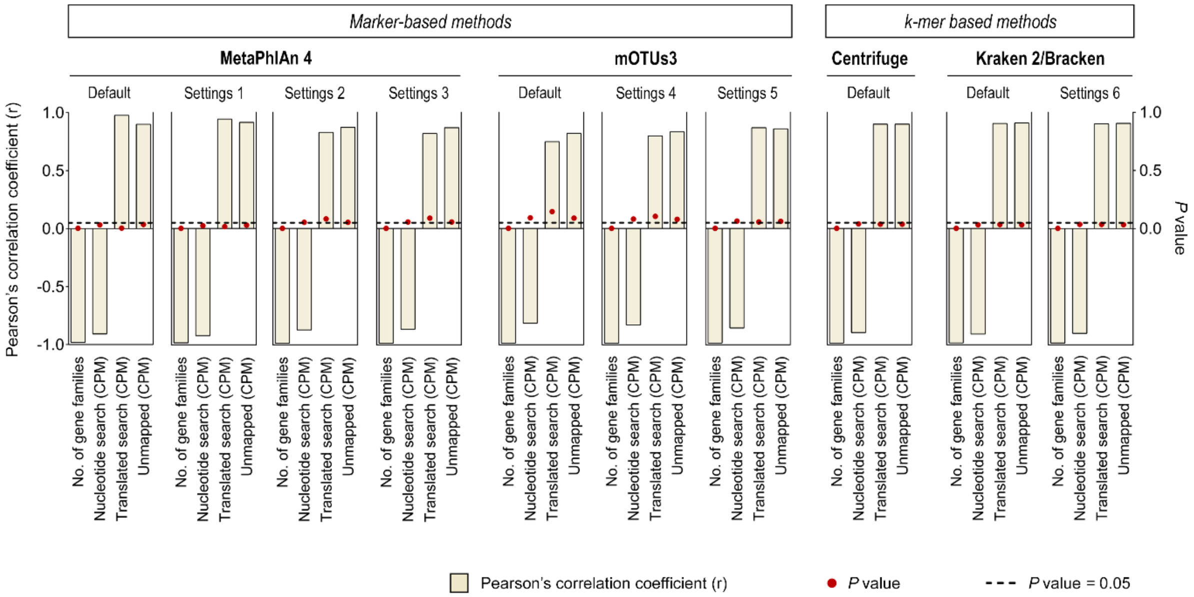

Supplementary Figure S3. Pearson's correlation between the proportion of host cells in synthetic samples and several variables in the functional analysis performed by HUMAnN 3 in combination with the taxonomic classifiers MetaPhlAn 4, mOTUs3, Centrifuge, and Kraken 2/Bracken (using different parameter settings). These variables include the number of gene families identified, the contribution of the nucleotide and translated searches (copies per million), and the abundance of unmapped (copies per million). Unmapped represents the reads that failed to map after both HUMAnN 3 alignment steps. Bars represent Pearson's correlation coefficient (r), red dots indicate the *P* value for each correlation, and dotted lines show the *P* value threshold below which the correlation is statistically significant (*P* = 0.05). CPM, copies per million.

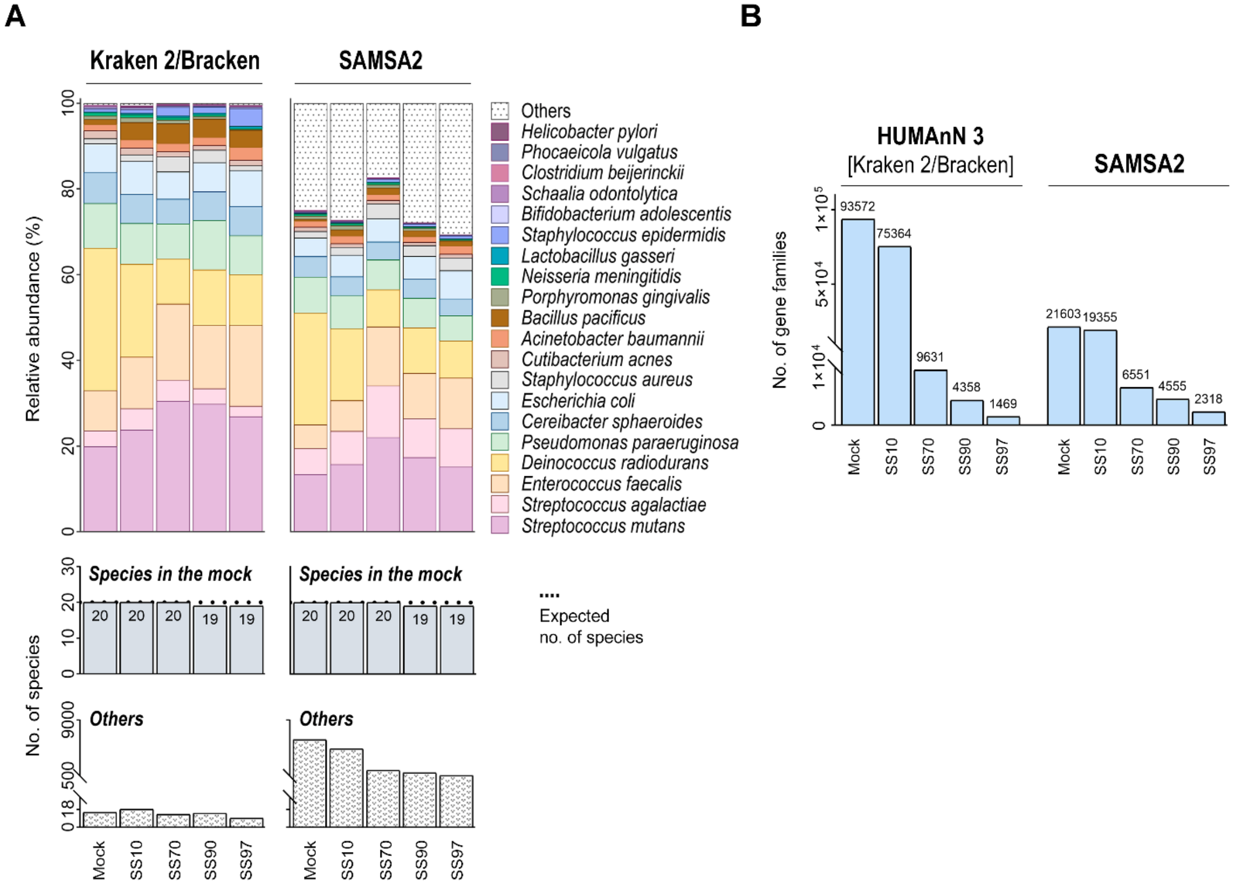

Supplementary Figure S4. Performance of HUMAnN 3 combined with optimized Kraken 2/Bracken compared to that of SAMSA2 pipeline. (A) Color-coded bar plots showing the taxonomic profiles at the species-level of the synthetic samples (SS) determined using Kraken 2/Bracken with optimized settings (left panel) and SAMSA2 (right panel). Others represent other microbial species identified that are not present in the mock community (false-positives). Bar plots showing the number of bacterial species of the mock community and the number of "Others" that the optimized Kraken 2/Bracken (left panel) and the SAMSA2 (right panel) were able to identify in the metatranscriptome of synthetic samples. Dotted line shows the expected number of species of the mock community in the samples. (B) Bar plots showing the number of gene families that HUMAnN 3 with optimized Kraken 2/Bracken (left panel) and SAMSA2 (right panel) were able to detect in the metatranscriptome of synthetic samples.
